# Field efficacy assessment of a combination of afoxolaner, moxidectin and pyrantel pamoate to treat dogs naturally infested with *Sarcoptes scabiei*

**DOI:** 10.1051/parasite/2026001

**Published:** 2026-01-22

**Authors:** Loïc Antoine, Elias Papadopoulos, Georgios Sioutas, Do Yew Tan, Maxime Madder, Eric Tielemans

**Affiliations:** 1 Boehringer Ingelheim Animal Health 69007 Lyon France; 2 Laboratory of Parasitology and Parasitic Diseases, School of Veterinary Medicine, Faculty of Health Sciences, Aristotle University of Thessaloniki 54124 Thessaloniki Greece; 3 Clinglobal Limited 90903 Tamarin Mauritius

**Keywords:** Afoxolaner, Efficacy, NexGard^®^ Plus, Sarcoptic mange, *Sarcoptes scabiei*

## Abstract

Canine sarcoptic mange, caused by *Sarcoptes scabiei*, is a highly contagious and intensely pruritic skin disease in dogs. It is prevalent worldwide and has zoonotic potential. Therefore, effective treatment is important to safeguard animal welfare and public health. The present clinical field study aimed to confirm the efficacy of NexGard^®^ Plus, an oral combination of afoxolaner, moxidectin and pyrantel pamoate, in treating dogs naturally infested with *S. scabiei*. It was a blinded, randomised, single-centre, negative-controlled efficacy study. Twenty naturally infested dogs were allocated into two groups: a group treated on Day 0 and Day 26/28 at the label dose, and an untreated control group. Skin scrapings were conducted similarly, once between Day −6 to 0, then on Days 26/28 and 56 for mite counts. Assessments of clinical signs were conducted at the same time intervals. In the treated group, mite infestations were reduced by 97% after the first treatment and were eliminated (100%) after the second treatment (*p* < 0.0005), while all dogs in the untreated control group remained infested for the whole study. Treated dogs had no pruritus, papules or crusts and clear evidence of hair regrowth by Day 56, unlike the dogs in the control group. This study demonstrated the elimination of *S. scabiei* mites and significant improvement of sarcoptic mange clinical signs in naturally infested dogs treated with the oral combination of afoxolaner, moxidectin and pyrantel.

## Abstract (fr)

La gale sarcoptique canine, causée par *Sarcoptes scabiei*, est une dermatose très contagieuse et extrêmement prurigineuse chez le chien. Elle est répandue dans le monde entier et présente un potentiel zoonotique. Un traitement efficace est donc essentiel pour préserver le bien-être animal et la santé publique. La présente étude clinique de terrain visait à confirmer l’efficacité de NexGard^®^ Plus, une association orale d’afoxolaner, de moxidectine et de pamoate de pyrantel, dans le traitement des chiens naturellement infestés par *S. scabiei*. Il s’agissait d’une étude d’efficacité monocentrique, randomisée, en double aveugle et contrôlée par un placebo. Vingt chiens naturellement infestés ont été répartis en deux groupes : un groupe traité aux jours 0 et 26/28 à la dose recommandée, et un groupe témoin non traité. Des prélèvements cutanés ont été effectués de la même manière, une première fois entre le jour −6 et le jour 0, puis aux jours 26, 28 et 56 pour le dénombrement des acariens. L’évaluation des signes cliniques a été réalisée aux mêmes intervalles. Dans le groupe traité, l’infestation par les acariens a diminué de 97% après le premier traitement et a été totalement éliminée (100 %) après le second (*p* < 0,0005), tandis que tous les chiens du groupe témoin non traité sont restés infestés pendant toute la durée de l’étude. Les chiens traités ne présentaient ni prurit, ni papules, ni croûtes, et une repousse des poils était clairement visible au jour 56, contrairement aux chiens du groupe témoin. Cette étude a démontré l’élimination des acariens *S. scabiei* et une amélioration significative des signes cliniques de la gale sarcoptique chez les chiens naturellement infestés traités par l’association orale d’afoxolaner, de moxidectine et de pyrantel.

## Introduction

Canine sarcoptic mange is a highly contagious and intensely pruritic skin disease, caused by the epidermal mite *Sarcoptes scabiei* var. *canis* (Linnaeus, 1758) [11, 16]. This parasitic infestation is observed worldwide in dogs, with a higher incidence in younger animals. Pet owners represent a key population at risk for zoonotic scabies, as dogs are the most frequently reported source of human infestations worldwide [18]. Following prolonged exposure, humans develop erythematous and papular lesions, but the infestation is usually self-limiting as the mites cannot reproduce on human skin, making humans a dead-end host [23]. Additionally, *S. scabiei* has been found in various other species, (*e.g.*, cats, raccoons, wombats and foxes) [7, 13, 29], highlighting its lack of host specificity and the risk for public health [7, 11, 16, 23]. Diagnosis of sarcoptic mange in dogs relies on the identification of mites through skin scrapings, which can be challenging due to the low sensitivity of the method [14, 19]. Clinical signs such as severe pruritus, erythematous rash, yellowish skin crusts, as well as clinical response to acaricidal treatment can support the diagnosis [16].

Current treatment options for sarcoptic mange include a variety of topical and systemic on- and off-label therapies. Historical topical treatments, such as amitraz dips and fipronil spray, act by contact on the skin surface, but can be influenced by factors like shampooing or bathing [8]. Systemic treatments, including macrocyclic lactones, like selamectin as well as moxidectin alone or in combination products, have proven to be effective against sarcoptic mange [10, 15, 22, 24, 28]. The more recent systemic isoxazolines compound have demonstrated high efficacy against canine sarcoptic mange and have become the preferred option due to their sustained acaricidal activity, reducing the re-infestation risk [4]. These compounds include afoxolaner [5, 12, 21], fluralaner [6, 20, 26], sarolaner[1, 2] and lotilaner [17].

Isoxazolines have also demonstrated efficacy against sarcoptic mange in other types of hosts, like afoxolaner for the treatment of scabies in pigs [3, 25], and fluralaner for the treatment of scabies in raccoons [13] and the bare-nosed wombat [29].

NexGard^®^ Plus (Boehringer Ingelheim) is an endectoparasiticide combination product for dogs, combining afoxolaner, moxidectin and pyrantel pamoate, which had never been used to treat canine sarcoptic infestations prior to this study. Therefore, the current clinical field study aimed to verify its efficacy in treating dogs naturally infested with *S. scabiei*.

## Materials and methods

### Ethics

Animals were managed with due regard for their wellbeing, and the study designs were reviewed and approved by the Sponsor and local Institutional Animal Care and Use Committees. The current trial was approved by the Ethics Committee of the Aristotle University of Thessaloniki (162009/2024).

This study, conducted in 2025 in Greece, was a blinded, randomised, single centre, negative-controlled study with a parallel group design. It was conducted in compliance with VICH GL9 “Good Clinical Practice” guidelines and Directive 2010/63/EU on the protection of animals used for scientific purposes.

### Study animals

Twenty client-owned dogs diagnosed positive for sarcoptic mange were recruited, mostly from rural areas and housed in animal facility kennels for the duration of the study. Dogs from each group were housed in a separate open kennel, in cages of 2 m × 2 m. Each cage accommodated 2 dogs of the same sex and treatment group. Outside of their pens, animals did not have physical contact with each other and did not share common places. Dogs were from various breeds and both sexes (12 females and 8 males). At baseline (Day −5), Group 1 had an average body weight of 12.3 kg, and Group 2 of 15.1 kg. They were healthy per clinical examination, except for clinical signs of sarcoptic mange, and were free of *Dirofilaria immitis*. Exclusion criteria were: dogs intended for breeding, pregnant or lactating females, dogs treated with glucocorticoids, macrocyclic lactones or ectoparasiticides within 12 weeks of first intended treatment, dogs treated for heartworm prevention, or with pre-existing pruritic skin conditions unrelated to sarcoptic mange, such as atopic dermatitis.

An informed consent and agreement form was completed and signed by the owners.

### Study design

**Figure 1:**
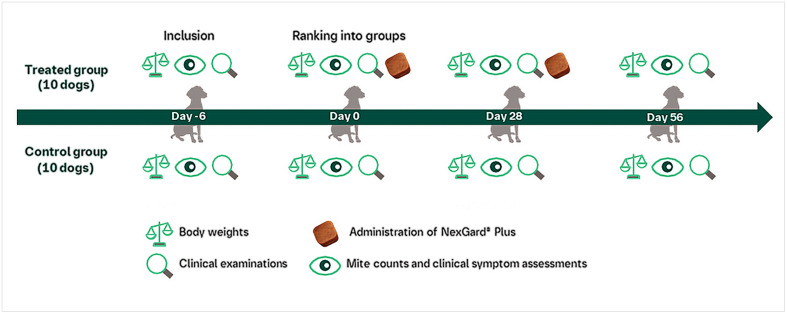
Study design.

The study followed a randomised block design, with ten blocks of two dogs based on pre-treatment live mite counts obtained during the acclimatisation period (Day −6 to Day 0). Within block, one dog was randomly allocated to the control group (Group 1) and to the treated group (Group 2), resulting in a total of 10 dogs in each group (Fig. 1).

Group 1: 10 untreated dogs.

Group 2: 10 dogs treated orally with NexGard^®^ Plus at label dose on Days 0 and 26/28. The actual afoxolaner administered doses ranged from 2.8 to 4.9 mg/kg on Day 0 and similarly 2.8 to 4.9 mg/kg on Day 28. Monthly moxidectin doses ranged from 13.4 to 23.7 μg/kg, concentrations that are insufficient for efficacy against sarcoptic mange (internal unpublished data).

### Study variables

#### Mite counts

The efficacy evaluation was primarily based on the reduction of *S. scabiei* mite numbers. Skin scrapings on a skin surface of ~4 cm^2^ were performed at baseline (Days −6 to 0) from 5 different body areas suspected of being infested (total surface = ~20 cm^2^). These areas were recorded on a silhouette for each individual animal, and scraping the same site multiple times at the next evaluations (Days 26/28 and 56) was avoided for animal welfare reasons. The scrapings were made with a scalpel blade until capillary oozing occurred and were examined under a stereomicroscope (Olympus, Research Stereomicroscope System SZH10, Hamburg, Germany) (8× to 64×) for the presence and count of live mites.

#### Clinical signs

The clinical signs were assessed and scored on the days when scrapings were made. They included erythematous papules, crusts or scales, and areas of hair loss. The presence or absence of pruritus was also documented over a 5-minute observation period.

### Data analysis

The experimental unit was the individual dog.

The first efficacy criterion was reduction in mite counts, evaluated following two different methods.

#### Mite-free dogs

The proportion of the total number of mite-free dogs in each of the two groups was the main efficacy criterion, calculated by:

$$\mathrm{Mite}-\mathrm{free}\;\mathrm{dogs}\;\left(\mathrm{MFD}\right)=\;\frac{\mathrm{Number}\;\mathrm{of}\;\mathrm{dogs}\;\mathrm{with}\;\mathrm{an}\;\mathrm{absence}\;\mathrm{of}\;\mathrm{live}\;\mathrm{mites}}{\mathrm{Total}\;\mathrm{number}\;\mathrm{of}\;\mathrm{dogs}}\;.$$

$$\mathrm{Failure}\;\mathrm{rate}\;\left(\mathrm{FR}\right)=\;1\;–\;\mathrm{MFD}.$$

$$\mathrm{Efficacy}\;(\%)\;=\;\left(1\;–\;\frac{\mathrm{FR}\;\mathrm{treated}\;\mathrm{group}}{\mathrm{FR}\;\mathrm{control}\;\mathrm{group}}\right)\times \;100.$$

#### Reduction relative to the control group:

Group comparisons on Days 26/28 and 56 were based on the number of live mites proportional to the number of live mites the animals were initially infested with, in their group. Least square means proportions of live mites were used for the calculations.

The percentage efficacy relative to the control group was calculated for the treated group at each assessment day using the following formula:

$$\mathrm{Percentage}\;\mathrm{efficacy}\;=100\;\times \frac{P_{c}-P_{t}}{P_{c}}.$$

Where:

*P*_*c*_ = Least square mean proportion of live mites (immature and adult) in the control group (Group 1) at a specific time point.

*P*_*t*_ = Least square mean proportion of live mites (immature and adult) in the treated group (Group 2) at a specific time point.

The second efficacy criterion was the resolution of the cutaneous clinical signs.

The presence or absence of pruritus, erythema, papules, crusts and area of hair loss by Day 56 were used to determine clinical response.

### Statistical methods

For the mite count, a generalised linear model with a treatment effect and a log link function for binomial distributed data was used. For all statistical analyses, SAS version 9.4 was used.

## Results

At the start of the trial, all dogs presented clinical signs consistent with sarcoptic mange, and at least one adult mite was identified in each dog. At the end of the study on Day 56, all 10 dogs in the negative control group were still positive for mite infestation and presented scaling and crusting, with mild to moderate erythema, indicating persistent mite infestation during the study.

The arithmetic mean of live mites in the control group was 5.6 at inclusion and increased to 19.6 on day 56 (Table 1).

**Table 1:** Results of mite counts.

	Control	Treated	% Reduction	*p*-value
Change from baseline within group				
Baseline (AM)	5.6	5.6		
Day 28 (AM)	18.5	1.1		
Day 56 (AM)	19.6	0		
Proportion of mite-free dogs				
% mite-free Baseline	0	0		
% mite-free Day 28	0	50		
% mite-free Day 56	0	100		<0.0001*
Group comparison using proportion of live mites relative to baseline				
Least square mean proportion of live mites Day 28	4.7	0.1	97.0	0.0026**
Least square mean proportion of live mites Day 56	4.7	0.0	100	0.0003**

* Fischer’s exact test.

** Linear mixed model with treatment as fixed effect and block as random effect.

In the treated group, the arithmetic mean number of mites was reduced from 5.6 at inclusion to 0 on Day 56. NexGard^®^ Plus was highly effective in treating mite infestation, with significant efficacy of 97% on Day 28 (*p* < 0.005) and 100% on Day 56 (*p* < 0.0005). On Day 56, all treated dogs were free of mites. No adverse events were observed during the course of the study.

At inclusion, in the treated group, 8/10 dogs had mild to moderate pruritus and scaling as well as mild to severe erythema, 5/10 dogs had mild to severe crusting, and 3/10 dogs had mild to moderate papules. By Day 56, all dogs in the treated group had complete absence of sarcoptic mange cutaneous signs. At the end of the study, all dogs in the control group received rescue treatment.

## Discussion

This is the first study demonstrating the efficacy of afoxolaner given orally in combination with moxidectin and pyrantel pamoate against *S. scabiei* mite infestation. Afoxolaner has previously demonstrated efficacy in experimental and field studies [5, 12]. Moxidectin has also shown efficacy, but only at doses much higher than those used in this study [15, 28]. Usually, isoxazolines administered monthly require 2 monthly doses to fully eliminate mites.

Clinical resolution of sarcoptic mange associated clinical signs, such as pruritus and skin lesions, and a reduction in the number of mites has previously been demonstrated with other active ingredients like selamectin [10, 15, 28], fipronil [27], fluralaner [6, 20, 26], lotilaner [17], and sarolaner [1, 2], despite some reported cases of mites refractory to treatment [27].

Skin scrapings were performed on Day 0, and both groups started the study with an average count of 5.6 live mites per dog. At Day 28, all dogs in the control group and 5 dogs out of 10 in the treated group had at least 1 live mite, but the average number of mites was significantly different, with an average count of 18.5 live mites per dog in the control group and only 1.1 live mites in the treated group. By Day 56, all dogs in the untreated group were infested with an average of 19.6 live mites, while all dogs in the NexGard^®^ Plus group were negative. The increasing level of infestation observed in the control group confirmed that sarcoptic mange did not self-cure, but on the contrary, worsened over time. This deterioration may be explained by the fact that infestation was recent in these dogs at the beginning of the study, and/or by other environmental changes (food, housing, local climate, increased environmental/cage contamination with mites, co-infections) over the two months of the study [9].

The significant reduction of mites on Day 28 in the treated group led to a total absence of pruritus, erythema, scaling, crusting or papules in 9 out of 10 dogs. Only mild hair loss was still visible, which was expected, considering the time necessary for hair regrowth. A single treated dog was observed at Day 28 with a relatively high number of live mites (7) after skin scraping associated with mild signs of pruritus, erythema, scaling, as well as moderate presence of crusts. This could be explained by heavier infestation at the start of the study and a lower immune status. At inclusion, this dog had a live mite count almost twice as high as the mean in the treated group (10 *vs.* 5.6). Nevertheless, the second dose administered on Day 28 achieved full antiparasitic efficacy by Day 56 for this dog and all others.

It was observed that the two groups progressed in different directions in terms of body weight, with an average body weight loss of 1.8% in the control group (9/10 dogs lost body weight) and an average body weight gain of 4.1% in the NexGard^®^ Plus treated group (all dogs gained body weight) on Day 56, in comparison to the baseline values. These trends may suggest a potential improvement in the general health of dogs treated with NexGard^®^ Plus, likely due to reduced pruritus and discomfort, combined with the possible deworming effect of the product, allowing for more effective feeding.

No abnormal clinical signs were observed during daily health observations and no adverse events were reported during the study, supporting the positive safety profile of NexGard^®^ Plus in dogs. This is particularly important, given that sarcoptic mange can affect dogs of all ages and health conditions, but displays higher prevalence in neglected younger dogs.

## Conclusion

The objective of the study was to demonstrate the efficacy of NexGard^®^ Plus in dogs naturally infested with *S. scabiei* in a clinical field study. Efficacy was confirmed with a 100% reduction in mite counts in the treated dogs, compared to baseline and to the control group, with full recovery from sarcoptic mange clinical signs by Day 56.
